# Acute Limb Ischemia: A Catastrophic COVID-19 Sequel Leading to Amputation

**DOI:** 10.7759/cureus.16456

**Published:** 2021-07-18

**Authors:** Jayanta Kumar Biswal, Sujit Kumar Mohanty, Satya Narayan Behera, Santanu Kumar Swain, Ashok Kumar Sahoo

**Affiliations:** 1 Surgery, Srirama Chandra Bhanja Medical College and Hospital, Cuttack, IND; 2 Surgery, Jawaharlal Institute of Postgraduate Medical Education & Research, Puducherry, IND

**Keywords:** covid-19, hypercoagulable, microangiopathy, macroangiopathy, amputation

## Abstract

A positive-sense single-stranded ribonucleic acid (RNA) virus causes the novel coronavirus illness 2019 (COVID-19). COVID-19 symptoms range from mild to moderate to severe and very severe. Fever, cough, headache, anosmia, ageusia, body ache, and diarrhoea are mild to moderate grade symptoms, whereas systemic involvements (pneumonia, myocarditis, stroke, and other coagulation abnormalities) are more serious. Except for a few patients who have mild complaints of cough and shortness of breath, the majority of patients are recuperating entirely from the viral infection. Patients with severe to very severe illnesses experience significant lung damage and fibrosis. These are the patients who are more likely to experience extrapulmonary complications after COVID-19. The disease's aberrant presentation may be related to systemic involvement and a hypercoagulable condition with micro and macro-angiopathy. Acute limb ischemia is one of the symptoms of the hypercoagulable condition. Its presentation can be in the form of chilblains, bullae, acral cyanosis, bruising, blood blisters, dry gangrene, or life-threatening acute limb ischemia. Unfortunately, most patients have to undergo amputation due to a delay in presentation or rapidly progressing disease. Here we present a case series of two COVID-19 infected patients who were initially discharged from the hospital after proper treatment but developed acute limb ischemia within the home isolation period and their treatment strategy.

## Introduction

The novel coronavirus disease 2019 (COVID-19) is caused by a positive-sense single-stranded ribonucleic acid (RNA) virus [1]. It was first detected in Wuhan, China, in December 2019 as declared by WHO, and it was declared a global pandemic in March 2020 [2]. The first case in India was detected in January 2020 in Kerala [3]. Though initially thought to affect only the respiratory system, many types of research, including this, demonstrate extrapulmonary involvement. Several studies reveal that the atypical presentation of the disease might be due to systemic involvement and hypercoagulable state with micro and macro-angiopathy [4]. One of the manifestations of the hypercoagulable state is acute limb ischemia. Its presentation can be in the form of chilblains, bullae, acral cyanosis, bruising, blood blisters, dry gangrene, or life-threatening acute limb ischemia [5]. A few patients who present early recover with medical management. However, unfortunately, most patients need an amputation due to delayed presentation or rapidly progressing disease. Some patients also succumbed due to sepsis. Here we present a case series of acute lower limb ischemia following COVID-19 infection and their treatment strategy. Informed consent was taken from both the patients included in the study.

## Case presentation

Case Report 1

A 45-year-old gentleman presented to the emergency department with blackish discolouration of the left lower limb for four days. It initially started at a digital level and gradually progressed up to the level of mid-thigh. This was associated with severe pain due to which he was unable to walk. He was diagnosed to have hypertension for the last eight years and was on oral antihypertensive medications regularly. He had no history of any cardiac diseases, diabetes, or transient ischemic attack. He did not have any history of smoking or any family history of atherosclerotic disease.

He tested COVID-19 RT-PCR positive 16 days back, for which he was admitted to a tertiary care hospital since he had difficulty in breathing. Initially, he was managed in the ward for one day and then shifted to ICU given clinical deterioration. His inflammatory markers like c-Reactive Protein (CRP), ferritin, D-dimer, and IL-6 were raised. During the ICU stay, he received steroids, antibiotics, anti-virals, and antithrombotics. He was shifted out of ICU to the ward after 10 days of ICU stay and then discharged from the hospital after two more days of observation. There was no specific history to explain the unfortunate event except for the recent history of COVID-19.

**Figure 1:**
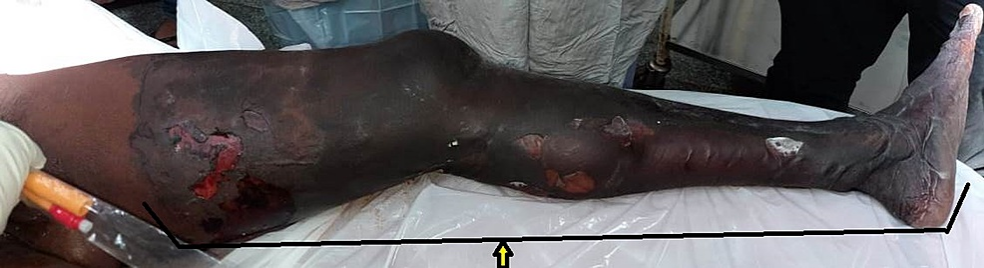
Acute limb ischemia of left lower limb up to mid thigh (Arrow)

At presentation, the patient was dehydrated and had tachycardia, but blood pressure was normal. The respiratory system examination revealed bilateral vesicular breath sound heard all over the chest without any added abnormal sound. The cardiovascular system examination was found to be normal. On local examination, wet gangrene of the left lower limb was present with the line of demarcation at the proximal thigh region (Figure 1).

**Figure 2:**
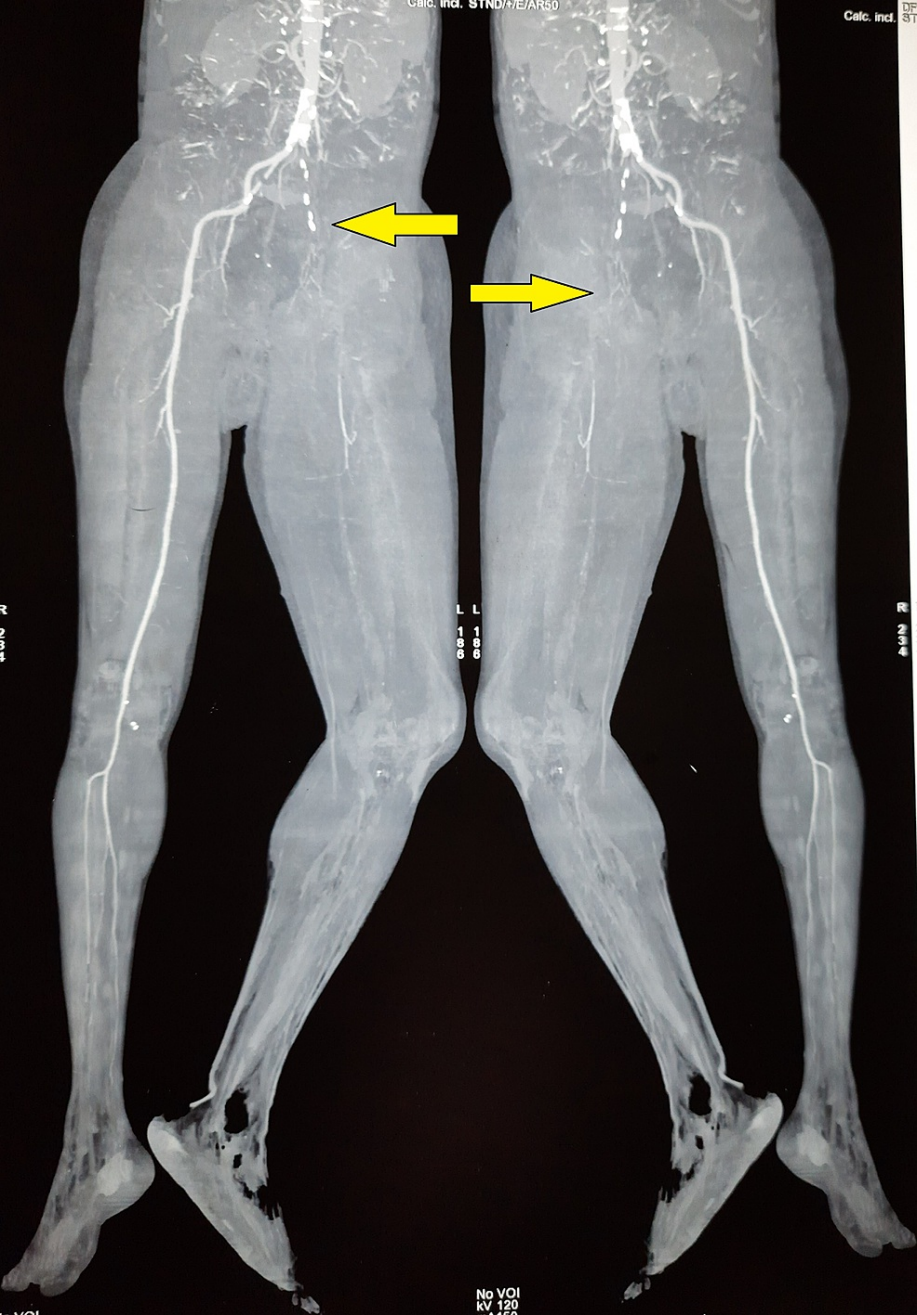
Maximum intensity projections of CTA of bilateral lower limbs showing complete occlusion of the blood supply to the left lower limb from the level of common iliac artery (Arrow). Poor collaterals noted. No distal reformation of the blood flow visualized. Also seen are the cellulitic changes in the left foot with loss of soft tissue. The blood supply to the right lower limb is preserved CTA = CT Angiography

**Figure 3:**
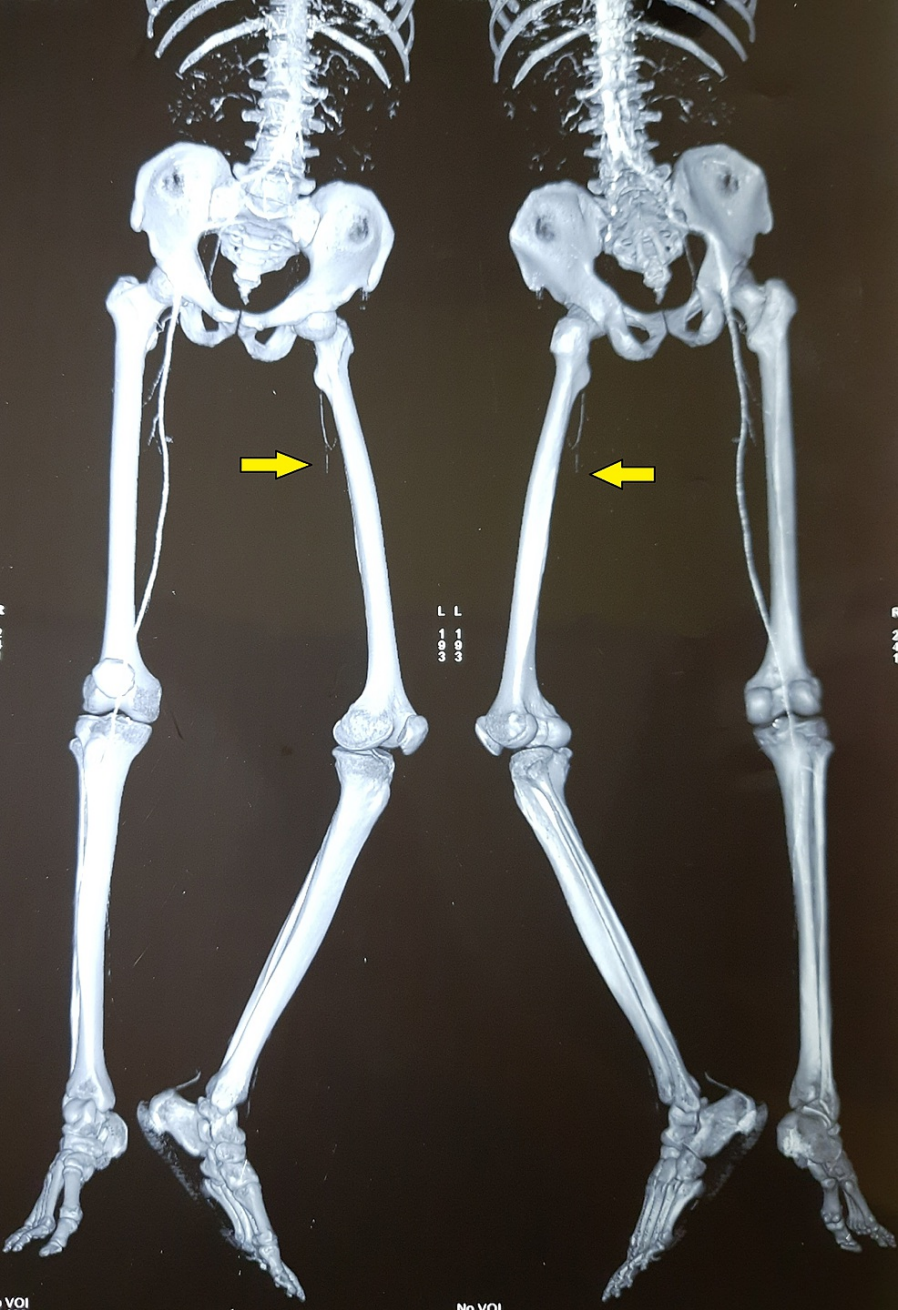
It is a surface shaded image of CTA depicting complete blockage blood supply to the left lower limb from the level of common iliac artery (Arrow) CTA= CT Angiography

There were few bullae present over the medial aspect of the thigh. There was tenderness and a local rise of temperature over the thigh region. The distal limb was cold to touch. All the pulses distal to femoral pulse were absent (popliteal, anterior and posterior tibial, and dorsalis pedis). There was a loss of motor and sensory activity below the knee. The patient was started on empirical broad-spectrum antibiotics and heparin injection. Subsequently, CT angiography was done, which revealed complete occlusion of the blood supply to the left lower limb from the level of the common iliac artery (Figure 2, 3).

**Figure 4:**
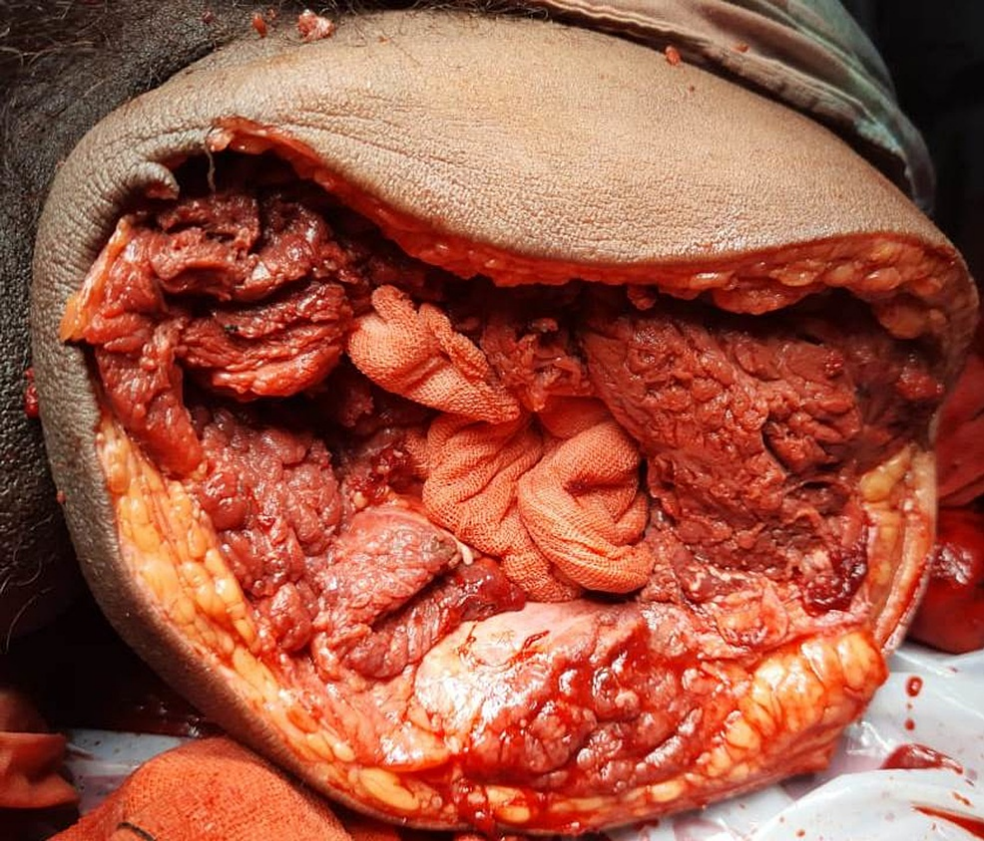
Intraoperative picture of left above-knee amputation with open wound

Echocardiography revealed no abnormality. Blood investigations showed neutrophilic leucocytosis with a deranged prothrombin time/ international normalized ratio (PT/INR). The lipid profile of the patient was found to be normal. Since gangrene was already established, the option of thrombolysis was ruled out. As the limb was non-salvageable, a left above knee amputation was planned (Figure 4).

Intraoperatively, the femoral artery and vein were found to be filled with thrombus at the level of amputation. Below the level of amputation, the muscles of the anterior and medial compartments were necrosed with no response to any stimulation and were having a putrid smell. The sciatic nerve and saphenous nerve were sloughed off and were felt like butter when touched. All posterior compartment muscles were seemed viable and had mild to moderate responses to stimulation. The stump was left open given poor vascularity, and the daily dressing was done. The wound was closed secondarily on a post-operative day five, following which the patient recovered without any complications and was discharged on post-operative day 13.

Case Report 2

Another patient, a 55-year-old gentleman, presented to the emergency department chief complaints of blackish discolouration of the left lower limb up to the knee for five days. It was started distally involving the toes at first, which gradually progressed to affect the limb up to the knee. He had no history of diabetes, cardiac disease, hypertension, or history of transient ischemic attack. There was no family history of any atherosclerotic disease. He was not a smoker. Notably, he was diagnosed as COVID-19 positive 21 days back. The inflammatory markers like CRP, ferritin, D-dimer, and IL-6 were raised. For this, he received treatment at a tertiary care hospital for 15 days with antibiotics, steroids and Anti-virals without anticoagulants (as he was categorised to have mild to moderate grade disease).

**Figure 5:**
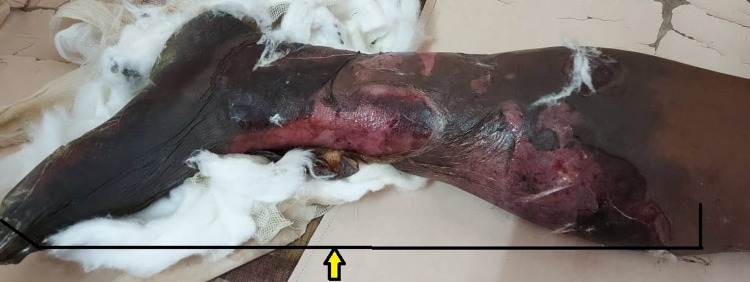
Acute limb ischemia of left lower limb up to knee (Arrow)

On examination, he had tachycardia and tachypnoea. The respiratory system examination revealed bilateral vesicular breath sound heard all over the chest without any added abnormal sound. The cardiovascular system examination showed no abnormality. Local examination of the left lower limb revealed gangrene below the knee joint (Figure 5).

**Figure 6:**
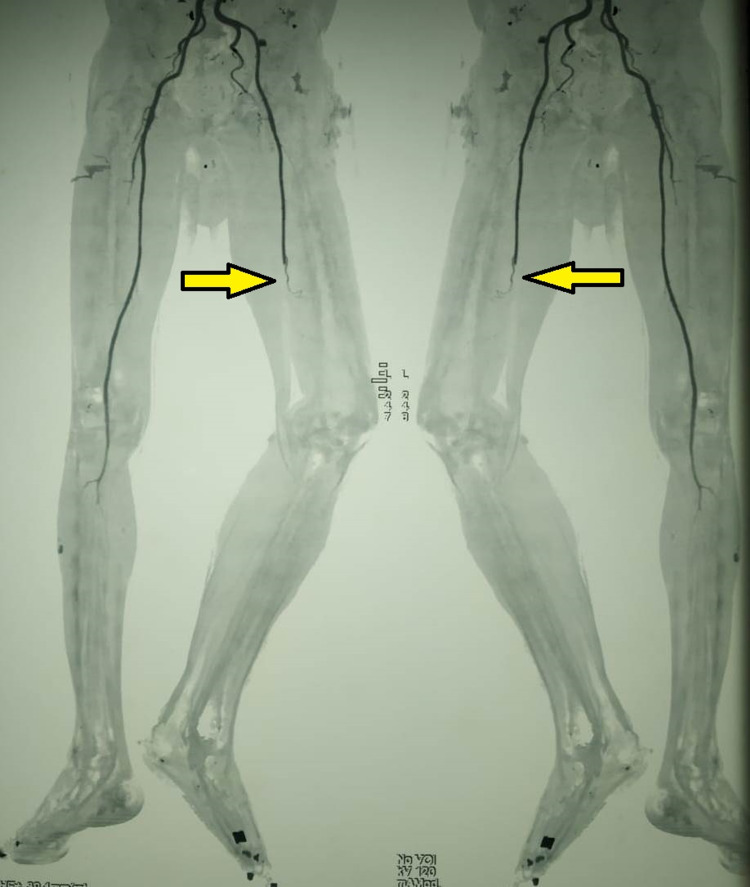
CT subtraction angiogram showing complete occlusion of the mid superficial femoral artery, popliteal artery, and infra popliteal artery with poor collateral status (Arrow)

**Figure 7:**
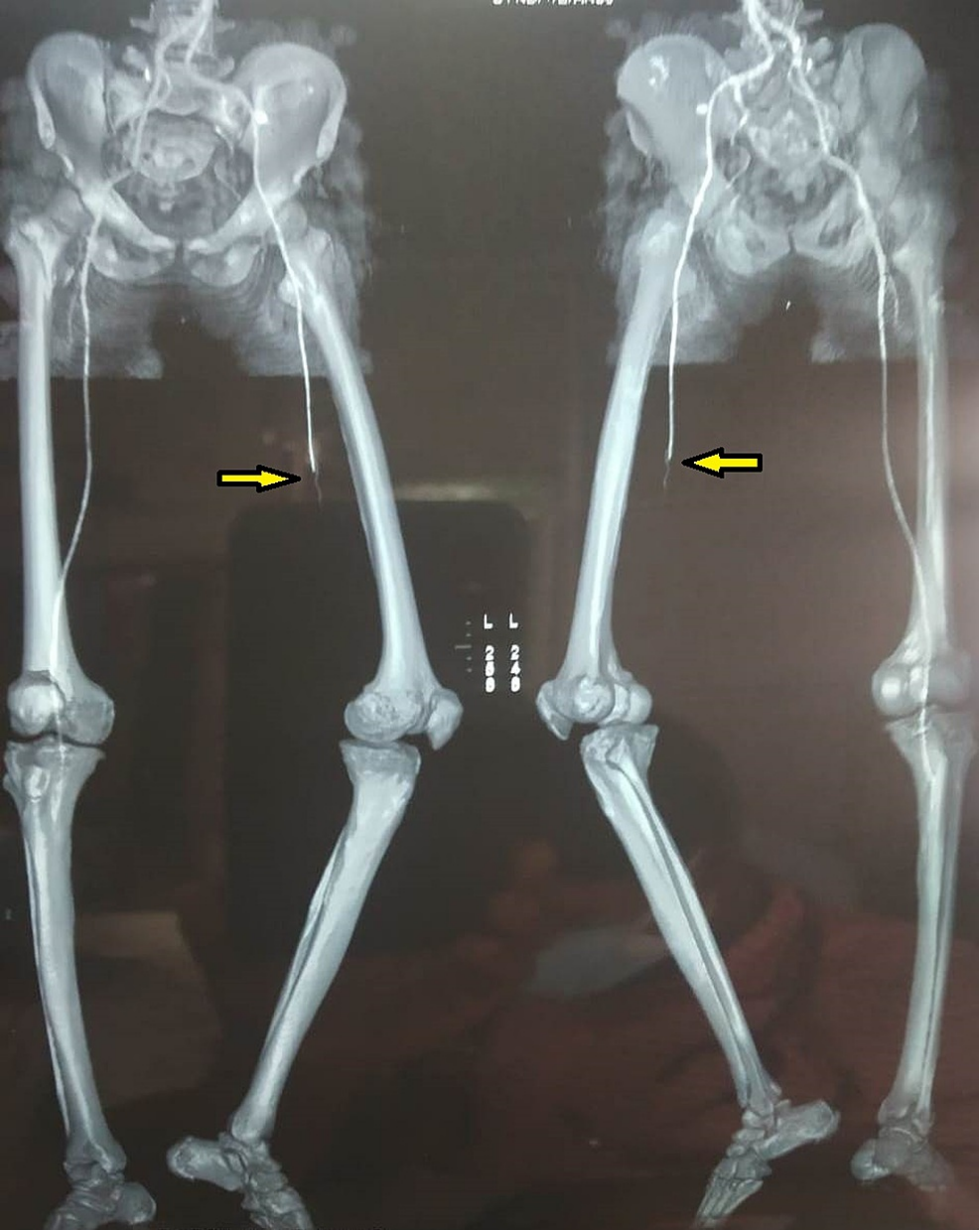
3D Virtual reconstructed CTA image showing complete blockage of the mid superficial femoral artery, popliteal artery, and infra popliteal artery with poor collateral status (Arrow). There is no distal reformation of the vessels CTA= CT Angiography

There was redness, edema, skin exfoliation, and tenderness up to the thigh. The foot distal to the ankle joint was cold. All the pulses were absent except the feeble left femoral pulse. The pulses in the opposite limb and other peripheral pulses were well felt. There was no motor activity below the knee. The patient was started on empirical broad-spectrum antibiotics. During the investigation, CT angiography showed complete occlusion of the mid superficial femoral artery, popliteal artery, and infra popliteal artery with poor collateral status (Figure 6, 7).

**Figure 8:**
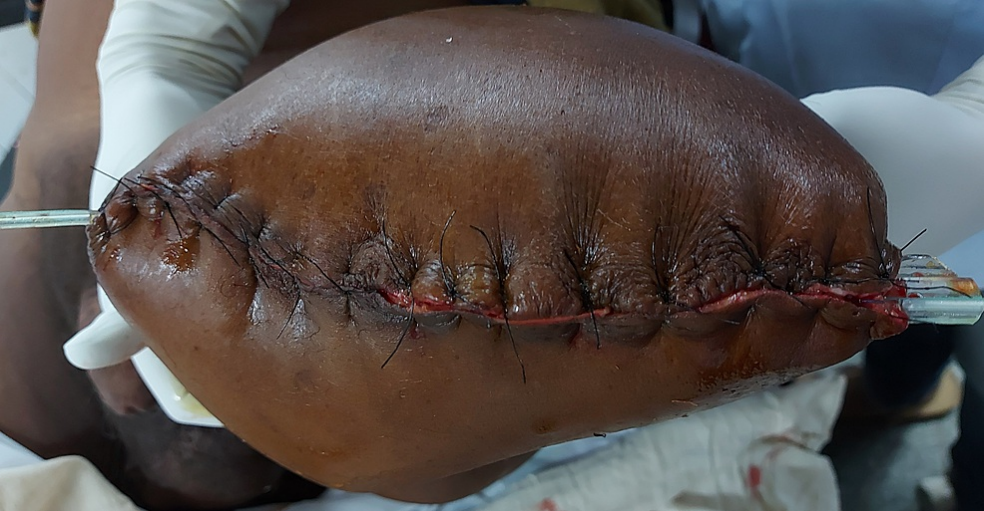
Intraoperative closed wound of left above knee amputation

The echocardiography of the patient was found to be normal. Blood investigations revealed neutrophilic leucocytosis. As there was already an onset of gangrene over the distal part of the limb, revascularisation was out of option. So the patient was left with no options other than left above knee amputation. Intraoperative findings revealed that the femoral artery and vein were filled with thrombus at the level of amputation. Below the level of amputation, the muscles of the anterior compartments were found to be gangrenous with no response to any stimulation with a putrid smell. The sciatic nerve and saphenous nerve were sloughed off and seemed like butter when touched. All posterior and medial compartment muscles appeared viable and had mild to moderate responses to stimulation. As the vascularity was adequate, the stump was closed primarily (Figure 8). The post-operative hospital stay was uneventful, and the patient was discharged on post-operative day 10.

## Discussion

The symptoms of COVID-19 are graded from mild to moderate to severe and very severe. While fever, cough, headache, anosmia, ageusia, body ache, diarrhoea constitute mild to moderate grade, systemic involvements (pneumonia, myocarditis, stroke, and other coagulation abnormalities) place patients in the severe grade of the disease. A large proportion of the patients are recovering completely after the viral infection, except a few having minor complaints of cough and shortness of breath. Patients presenting with severe to very severe diseases usually have significant lung damage and fibrosis [6]. These are the patients who are at higher risk of developing extra pulmonary post COVID complications. One school of thought is that the virus affects the heart, kidney, small intestine, testes, and vascular endothelium through angiotensin-converting enzyme 2 (ACE2) receptors [7]. Another group of investigators hypothesised that the prothrombotic state is caused directly by the virus damaging the endothelial cells through ACE-2 receptors of alveoli leading to endothelial cell activation and dysfunction [8]. Among the various manifestations of the COVID-19 infection, the prothrombotic effects of this virus are now taking a toll worldwide. Various studies have documented venous thromboembolic events and multiple arterial thrombotic manifestations ranging from lower extremity arterial occlusion to stroke [9]. The virus persuades an inflammatory cascade, leading to a prothrombotic state, causing microvascular and macrovascular endothelial damage [10]. In addition to this, inhibition of fibrinolysis exaggerates the thrombotic event. Liver secrets fibrinogen and thrombopoietin by the stimulation of interleukin 6 and damages the endothelium by activating the extrinsic pathway of the coagulation cascade [11]. A Dutch study revealed that patients with severe COVID-19 infection and admitted to ICU have a 31% increased risk of thrombotic complications [12]. Several studies showed that even after treating the severe COVID-19 patients with prophylactic or therapeutic antithrombotic therapy, the percentage of thrombotic complications is still high [13]. One of our patients was admitted to ICU and had a long ICU stay with prolonged antithrombotic therapy. Still, he developed limb ischemia on the 13th day after detection of COVID-19, though he was on antithrombotic agents. Other patient had mild to moderate disease and was not given any antithrombotic. He complained of blackish discolouration of the lower limb on the 11th day post-COVID-19 detection. So it remains a dilemma that how to prevent these catastrophic thrombotic events?

Another enthralling finding in both of our cases was a butter-like feeling of the sciatic nerve sheath. An Italian study by Zanin et al. revealed that the novel coronavirus could cause extensive nervous system demyelination [14]. This may be the explanation of our intraoperative findings. As more and more data regarding the post-COVID-19 thrombotic complications are getting surfaced worldwide, the challenges to treat this catastrophic event are also becoming tougher. A single-institution study done by Klok et al. showed the cumulative incidence of arterial or venous complications was around 57% [12]. Perini et al. reported four patients with post-COVID-19 acute limb ischemia [15]. Another Italian study revealed 20 patients with acute limb ischemia as an extrapulmonary post-COVID-19 thrombotic complication [16]. We are reporting these two post-COVID-19 acute lower limb ischemia as index cases in one of the largest tertiary care hospitals in Eastern India.

## Conclusions

Both patients in our series of two cases of acute lower limb ischemia were discharged following complete treatment of primary COVID-19 infection. It was the period of home isolation after discharge that led to the complication. The delay in seeking medical help cost both the patients their limbs. The patient's leg probably could have been saved if he had presented early. There is no established guideline for patients recovering from COVID-19 infection. Therefore, keeping the potentially debilitating effects of gangrene in mind, all patients should be seen as soon as possible if they recognise any signs or symptoms of limb ischemia (tingling, numbness, or discolouration). As one of the COVID-19 extrapulmonary manifestations, limb ischemia may present in the emergency department. Therefore, the history of COVID-19 should be taken into account when evaluating these patients. There has been no specific guideline developed to prevent or manage thrombosis complications. It remains a dilemma whether to use or not to use antithrombotics. If antithrombotics are to be used, who will receive them? Additional research may shed light on these unanswered questions.

## References

[1] Alattar KO, Subhi FN, Saif Alshamsi AH, Eisa N, Shaikh NA, Mobushar JA, Al Qasmi A (2021). COVID-19-associated leukocytoclastic vasculitis leading to gangrene and amputation. IDCases.

[2] Nabil A, Uto K, Elshemy MM, Soliman R, Hassan AA, Ebara M, Shiha G (2020). Current coronavirus (SARS-CoV-2) epidemiological, diagnostic and therapeutic approaches: an updated review until June 2020. EXCLI J.

[3] Andrews MA, Areekal B, Rajesh KR (2020). First confirmed case of COVID-19 infection in India: a case report. Indian J Med Res.

[4] Abou-Ismail MY, Diamond A, Kapoor S, Arafah Y, Nayak L (2020). The hypercoagulable state in COVID-19: incidence, pathophysiology, and management. Thromb Res.

[5] Zhang Y, Cao W, Xiao M (2020). Clinical and coagulation characteristics in 7 patients with critical COVID-2019 pneumonia and acro-ischemia [Chinese]. Zhonghua Xue Ye Xue Za Zhi.

[6] Kommoss FK, Schwab C, Tavernar L (2020). The pathology of severe COVID-19 related lung damage. Dtsch Arztebl Int.

[7] Hamming I, Timens W, Bulthuis ML, Lely AT, Navis G, van Goor H (2004). Tissue distribution of ACE2 protein, the functional receptor for SARS coronavirus. a first step in understanding SARS pathogenesis. J Pathol.

[8] Iwasaki M, Saito J, Zhao H, Sakamoto A, Hirota K, Ma D (2021). Inflammation triggered by SARS-CoV-2 and ACE2 augment drives multiple organ failure of severe COVID-19: molecular mechanisms and implications. Inflammation.

[9] Levi M, Thachil J, Iba T, Levy JH (2020). Coagulation abnormalities and thrombosis in patients with COVID-19. Lancet Haematol.

[10] Sypniewska G (2007). Pro-inflammatory and prothrombotic factors and metabolic syndrome. EJIFCC.

[11] Anwar S, Acharya S, Shabih S, Khabut A (2020). Acute limb ischemia in COVID-19 disease: a mysterious coagulopathy. Cureus.

[12] Klok FA, Kruip MJ, van der Meer NJ (2020). Incidence of thrombotic complications in critically ill ICU patients with COVID-19. Thromb Res.

[13] Helms J, Tacquard C, Severac F (2020). High risk of thrombosis in patients with severe SARS-CoV-2 infection: a multicenter prospective cohort study. Intensive Care Med.

[14] Zanin L, Saraceno G, Panciani PP, Renisi G, Signorini L, Migliorati K, Fontanella MM (2020). SARS-CoV-2 can induce brain and spine demyelinating lesions. Acta Neurochir (Wien).

[15] Perini P, Nabulsi B, Massoni CB, Azzarone M, Freyrie A (2020). Acute limb ischaemia in two young, non-atherosclerotic patients with COVID-19. Lancet.

[16] Bellosta R, Luzzani L, Natalini G (2020). Acute limb ischemia in patients with COVID-19 pneumonia. J Vasc Surg.

